# Longitudinal association between tobacco use and the onset of depressive symptoms among Swedish adolescents: the Kupol cohort study

**DOI:** 10.1007/s00787-018-1237-6

**Published:** 2018-10-12

**Authors:** Elena Raffetti, Francesco Donato, Yvonne Forsell, Maria Rosaria Galanti

**Affiliations:** 1 Department of Public Health Sciences, Karolinska Institutet, Solnavägen 1 E, 113 65 Stockholm, Sweden; 2 Unit of Hygiene, Epidemiology and Public Health, University of Brescia, Viale Europa 11, 25123 Brescia, Italy; 3 Centre for Epidemiology and Community Medicine, Stockholm Health Care District, Stockholm County Council, Stockholm, Sweden

**Keywords:** Tobacco exposure, Depression, Adolescence, Gender difference

## Abstract

Tobacco use in adolescence has been linked to the onset of depressive symptoms, but results of previous studies are inconsistent. The aim of this study was to clarify if tobacco use during early adolescence may affect the short-term onset of depressive symptoms. The study is based on Swedish Kupol study (3959 students). Current cigarette smoking, snus use, and tobacco dependence were assessed using questionnaires at baseline and 1-year follow-up. Outcome was the onset of depressive symptoms measured with the CES-DC scale, using a cut-off ≥ 30 as threshold. Adjusted linear and logistic regression models were employed to calculate odds ratios (OR) and corresponding 95% confidence intervals (CI). CES-DC mean score at baseline was 14.3, higher in females than males (17.5 vs 10.9). The incidence of depressive symptoms at follow-up was 8.3%, greater in current than never smokers at baseline (13.7% vs 3.1%). Current cigarette smoking at the age of 13 years was strongly associated with the onset of depressive symptoms 1 year later, particularly in males (OR 12.7, 95% CI: 2.5–63.9), with a significant interaction between tobacco use and sex; feeling dependent on tobacco was also associated with depressive symptoms in males but not in females. Snus and overall tobacco use were not associated with the onset of depressive symptoms. Tobacco use during adolescence appears to influence the onset of depressive symptoms, with a stronger association in males than females. Pubertal maturation and sex-specific response patterns to the scale instrument may explain the moderating effect of sex.

**Electronic supplementary material:**

The online version of this article (10.1007/s00787-018-1237-6) contains supplementary material, which is available to authorized users.

## Introduction

Adolescence is a pivotal time period in brain development when life experiences and environmental factors greatly influence the remodeling of the synaptic circuits. Furthermore, adolescents begin to use tobacco more during this age than at any other developmental stages [1]. Several studies have described the comorbidity between tobacco dependence and depressive symptoms in both adults and teenagers [2–4]. This robust relationship between tobacco dependence and depressive symptoms could be due to: (1) shared risk factors for both conditions; (2) the use of tobacco as a self-medication for relieving depressive mood (tobacco use as consequence of sub-clinical or clinical depressive symptoms) and (3) tobacco being a risk factor for the development of depressive symptoms (depressive symptoms as a consequence of tobacco use).

The two latter associations were examined in a recent systematic review [4]. The results of the included studies varied substantially, with evidence for positive associations in both directions as well as null findings. The inconsistence of findings across studies may be explained by the heterogeneity of populations, different follow-up lengths and the assessment of the onset of depressive symptoms. The causal role of tobacco use in determining the onset of depressive symptoms [pathway (3) above] is the most relevant question in the public health domain. This determinism is biologically plausible. In fact, it has been postulated that nicotine in smoking alters neurotransmitter pathways in the brain, possibly leading to the onset of depressive symptoms [5, 6]. Further, it has been hypothesized that the effect of cigarette smoking could be mediated by increased cortisol levels, mimicking a hormonal condition similar to chronic stress. Indeed cigarette smoking seems to activate the hypothalamic–pituitary–adrenal axis increasing the cortisol awakening response [7].

Explanations of a potential causal relationship between tobacco use and depressive moods, should consider four points: (1) the neurobiology of adolescent brain and (2) its sex differences; (3) the latency between exposure and onset of symptoms, and (4) patterns of exposure tobacco toxicants. Furthermore, it would be important to investigate the short-term association of tobacco use with depressive symptoms incidence in order to reduce the likelihood of other potentially causal exposures (e.g., stressful life events). Sex-specific patterns of association are also of interest because of the higher susceptibility of females compared to males to both depressive mood and the adverse effects of tobacco [8, 9]. Therefore, it can be hypothesized that the association between tobacco use and depressive symptoms if any would be stronger among females. Finally, regular smoking rather than initial episodes of smoking should be the exposure of interest, as the latter is often consisting in just a few puffs, therefore unlikely to determine the postulated alteration of neurotransmitter pathways and the subsequent development of depressive mood [5, 10].

To the best of our knowledge, there is no longitudinal study in Sweden exploring this association. A peculiarity of the Swedish context is the wide use of “snus” among men and boys. Snus is a variety of moist smokeless tobacco product [11] which can be thought of as a nicotine delivery device devoid of toxicants connected with burned tobacco. To analyze this type of tobacco use in relation to juvenile depressive symptoms may prove important in elucidating the potential biological mechanisms behind the association. In fact, similar associations between cigarette smoking and snus use would support the plausibility of an effect mediated by nicotine rather than by other toxicants.

This study therefore aimed to contribute to the understanding of a potential causal association between tobacco use and the short-term onset of depressive symptoms among early adolescents. Also, we aimed to elucidate whether this association differed by sex and type of tobacco.

## Methods

### Kupol cohort

The Kupol study is a prospective cohort study in Sweden set up with the main purpose to investigate youth’s mental health in relation to school-level factors [12]. Students’ mental health and substance use have been assessed by self- completed questionnaires. Further information collected at the students’ level included general health, as well as family and school relationships. The students’ parents answered questionnaires with general demographic information as well as a rating of their children’s mental health. The Kupol cohort at baseline consists of 3959 children (48.2% boys) attending the 7th grade of compulsory school (13–14 years old).

The study was conducted in accordance with the guidelines of the Declaration of Helsinki and the principles of Good Clinical Practice. Written informed consent was obtained from all parents or legal guardians. The study protocol was approved by the Ethic Board of the Stockholm Region (reference number: 2012/1904-31/01).

### Study population

The study population in this analysis includes participants in the Kupol cohort who answered the questionnaire at baseline and at a 1-year follow-up; also, information from at least one parental survey (baseline or a 1-year follow-up) was available.

### Exposure variables

Current tobacco use was assessed by self-report at both baseline and follow-up. Current smoking was categorized as any answer higher than 0 to the question: “On how many days of the past 30 days did you smoke cigarettes?”. Current snus use were defined in a similar way from the questions: “On how many of the past 30 days did you use snus?”. Likewise, current tobacco use was defined as any use of either cigarette or snus in the past 30 days. Perceived tobacco dependence was considered if an affirmative answer was given to the question: “Did you ever feel you are/were addicted to tobacco?”.

### Outcome variables

Depressive symptoms were assessed through the CES-DC (Center for Epidemiologic Studies Depressive symptoms Scale for Children) scale, a 20-item self-report depressive symptoms scale, widely used in the adolescent surveys [13–15]. The CES-DC score was considered both as continuous and dichotomous variable, this latter using the cut-off score ≥ 30 as indicative of depressive symptoms. We also evaluated depressive moods using the self- and parent-reported internalizing score of the Strengths and Difficulties Questionnaire (SDQ) [16], calculated as the sum of the emotional and peer problems subscales. Self-reported SDQ was dichotomized using a cut-off score ≥ 9 as indicative of high internalizing mental problems, while parent-reported using a cut-off score ≥ 8 [17].

### Other covariates

In order to adjust for potential confounding from familiar characteristics, we also retrieved from the parental survey information on parental education and birthplace.

### Statistical analysis

For descriptive analysis of the participants at baseline the Chi-square statistic was used to test for differences in the distribution of demographic and behavioral categorical variables between males and females, and Student’s *t* test was used to assess mean differences in continuous variables. Several approaches of analysis were employed in order to study the association between tobacco use and depressive symptoms. In a first step, we used linear regression models to explore current tobacco use or self-reported tobacco dependence at baseline in relation to the modification of the CES-DC score as a continuous variable between baseline and 1-year follow-up. This analysis included all eligible participants (step 1).

Secondly, we restricted the analysis to participants who at baseline scored on CES-DC below the threshold indicative of depressive symptoms, in order to evaluate the longitudinal association between current tobacco use or dependence and the onset of depressive symptoms 1 year later. In this analysis, CES-DC score was used as a dichotomous variable using logistic regression models (step 2).

Third, we restricted the analysis to participants who at baseline were both below the CES-DC threshold for depressive symptoms and reported no current use of or dependence on tobacco, in order to analyze the concurrent association between initiation of regular tobacco use or onset of tobacco dependence and onset of depressive symptoms.

For all analyses, results from regression models are reported: (1) unadjusted; (2) adjusted for CES-DC score at baseline; (3) as (2) further adjusted for sex, alcohol consumption, parental education, parental birthplace (step 3).

In addition, separate analyses were conducted according to sex and type of tobacco (cigarette smoking, snus or any tobacco). To perform a formal test of interaction between sex and tobacco use in the association with depressive symptoms, we also fitted fully adjusted logistic models with an interaction term.

In a sensitivity analysis we re-examined the relationship between current tobacco use or dependence and onset of depressive moods as in step 2 and step 3 using SDQ internalizing scores rather than CES-DC. Besides, we included subjects below the SDQ threshold for high internalizing problems (step 4).

The results of linear regression models and logistic regression models are reported as coefficients and odds ratios (ORs), respectively, together with their 95% confidence intervals (95% CIs).

All statistical tests were two-sided, assumed a level of significance of 0.05. All statistical analyses were performed using Stata software version 14.0 (StataCorp, College Station, TX, USA).

## Results

Among the 3275 students who answered the questionnaire at both baseline and 1-year follow-up, 80 individuals were excluded due to missing data on the depressive symptoms scale at baseline or at follow-up, leaving 3195 participants for the analysis. Of these, 51.2% were females, 70.8% had at least one parent with a university degree while 19.8% had at least one parent born outside Sweden (Table 1**)**. The prevalence of current tobacco use was low: 2.0% were current smokers, 0.8% current snus users, 2.6% were users of any tobacco and 1.7% reported having felt dependent on tobacco. The proportion of current cigarette smoking was higher among females than among males, whereas the proportion of snus users was significantly higher among males. The proportion of students consuming alcohol at least once a month was also low (males: 1.8%, females: 1.5%). The mean CES-DC score among the students who did not have high depressive symptoms at baseline was 14.3, with higher score among females than among males, 17.5 and 10.9 (*p* < 0.001), respectively.

**Table 1:** Students’ demographics, substance use and depressive symptoms score at baseline, the Kupol study 2013–2014

	Total *n* (%)	Males *n* (%)	Females *n* (%)	*p* value
Current cigarette smoking				
Yes	64 (2.0)	17 (1.1)	47 (2.8)	0.001
No	3130 (98.0)	1511 (98.9)	1619 (97.2)	
Current snus use				
Yes	26 (0.8)	22 (1.4)	4 (0.2)	< 0.001
No	3169 (99.2)	1507 (98.6)	1662 (99.8)	
Current tobacco use				
Yes	83 (2.6)	34 (2.2)	49 (2.9)	0.203
No	3112 (97.4)	1495 (97.8)	1617 (97.1)	
Self-reported tobacco dependence				
Yes	53 (1.7)	22 (1.5)	31 (1.9)	0.349
No	3094 (98.3)	1485 (98.5)	1609 (98.1)	
Alcohol consumption				
≥ Once a month	51 (1.6)	27 (1.8)	24 (1.5)	0.457
< Once a month	3118 (98.4)	1487 (98.2)	1631 (98.5)	
At least one parent with university education				
Yes	2247 (70.5)	1071 (70.3)	1176 (70.8)	
No	938 (29.5)	453 (29.7)	485 (29.2)	0.745
At least one parent born abroad				
Yes	582 (19.0)	268 (18.2)	314 (19.8)	0.274
No	2475 (81.0)	1202 (81.8)	1273 (80.2)	
Mean CES-DC (SD)	14.3 (10.2)	10.9 (7.6)	17.5 (11.1)	< 0.001

### The association between current tobacco use at baseline and CES-DC depressive symptoms score at follow-up (Table 2) (step 1)

Mean CES-DC score at 1-year follow-up was 15.7, again with higher values in females than males (19.5 vs 11.6, *p* < 0.001). Table 2 reports the mean CES-DC score at follow-up as a function of tobacco use at baseline, by sex and type of tobacco. Current smokers at baseline (*n* = 64) had a higher mean CES-DC score at follow-up compared to non-smokers (26.4 vs 15.5, respectively). This difference was found in both males (16.9 vs 11.5) and females (29.8 vs 19.2). The association was attenuated after adjustment for depressive symptoms score at baseline. In fully adjusted models including socio-demographic co-variates in addition to depressive symptoms score at baseline, the positive association of smoking with CES-DC score was confirmed in the whole cohort with a mean score difference of 3.4 as well as among males (mean score difference 4.4). However, the statistical interaction between sex and current smoking was not significant (*p* = 0.650) (data not shown).

**Table 2:** CES-DC score (continuous) at 1-year follow-up according to tobacco use at baseline, by sex and type of tobacco, the Kupol study 2013–2015

	*n*	CES-DC at follow-up Mean (SD)	Unadjusted model		Adjusted model A		Adjusted model B	
			Coeff. (95% CI)	*p* value	Coeff. (95% CI)	*p* value	Coeff. (95% CI)	*p* value
Current cigarette smoking								
Total students								
Yes	64	26.4 (15.3)	11.4 (8.8,14.0)	< 0.001	2.5 (0.3,4.6)	0.024	3.4 (1.0,5.7)^a^	0.006
No	3130	15.5 (10.6)						
Males								
Yes	17	16.9 (12.9)	7.2 (3.4,10.9)	< 0.001	3.2 (− 0.2,6.6)	0.571	4.4 (0.5,8.3)	0.028
No	1511	11.5 (7.9)						
Females								
Yes	47	29.8 (14.7)	10.7 (7.4,13.9)	< 0.001	1.8 (− 1.0,4.6)	0.199	3.0 (− 0.3,6.2)	0.072
No	1619	19.2 (11.3)						
Current snus use								
Total students								
Yes	26	15.7 (10.8)	− 0.6 (− 4.7,3.6)	0.781	− 2.0 (− 5.3,1.3)	0.226	− 0.1 (− 3.6,3.3)^a^	0.934
No	3169	15.1 (11.2)						
Males								
Yes	22	13.9 (10.6)	2.4 (− 1.0,5.8)	0.167	0.9 (− 2.1,3.8)	0.571	1.4 (− 1.8,4.6)	0.391
No	1507	11.5 (8.0)						
Females								
Yes	4	21.5 (14.0)	2.0 (− 9.3,13.3)	0.727	− 6.4 (− 15.5,2.7)	0.170	− 6.4 (− 15.6,2.8)	0.172
No	1662	19.5 (11.5)						
Current tobacco use								
Total students								
Yes	83	23.2 (15.1)	8.2 (5.9,10.5)	< 0.001	0.8 (− 1.1,2.7)	0.405	1.9 (− 0.2,4.0)^a^	0.073
No	3112	15.5 (10.6)						
Males								
Yes	34	14.8 (10.9)	4.2 (1.5,6.9)	0.002	1.0 (− 1.4,3.4)	0.424	1.8 (− 0.9,4.4)	0.195
No	1495	11.5 (7.9)						
Females								
Yes	49	29.0 (14.9)	9.9 (6.7,13.1)	< 0.001	1.1 (− 1.5,3.8)	0.401	2.0 (− 1.1,5.1)	0.207
No	1617	19.2 (11.3)						
Self-reported tobacco dependence								
Total students								
Yes	53	26.2 (15.2)	10.3 (7.4,13.2)	< 0.001	2.7 (0.3,5.0)	0.025	3.4 (0.9,6.0)^a^	0.008
No	3094	15.5 (10.6)						
Males								
Yes	22	19.0 (14.1)	6.8 (3.5,10.1)	< 0.001	5.6 (2.6,8.5)	< 0.001	7.3 (4.0,10.6)	< 0.001
No	1485	11.5 (7.8)						
Females								
Yes	31	31.4 (14.0)	12.2 (8.1,16.3)	< 0.001	1.1 (− 2.3,4.5)	0.518	0.3 (− 3.5,4.1)	0.876
No	1609	19.2 (11.4)						

^a^Model A adjusted for CES-DC score at baseline. Model B as model A further adjusted for alcohol consumption, parental education, parental birthplace and sex

In adjusted models current snus use at baseline (*n* = 26) was not associated with depressive symptoms score at follow-up, similarly to current use of any tobacco (*n* = 83).

Participants who referred having felt dependent on tobacco (*n* = 53) had a higher CES-DC score at follow-up than those non-dependent, 26.2 vs 15.5, respectively. After adjustment for baseline CES-DC alone or in addition to other co-variates perceived tobacco dependence at baseline significantly predicted CES-DC score at follow-up in the whole cohort (mean difference = 3.4) and among males (mean difference = 7.3), but not among females. The interaction between sex and tobacco dependence was statistically significant (*p* = 0.017) in the fully adjusted regression model.

### Association between current tobacco use at baseline and incidence of depressive symptoms at follow-up (Table 3) (step 2)

Among 2900 students who at baseline scored below the conventional score threshold of 30 on CES-DC, the incidence of depressive symptoms (score at or above 30) after 1 year was 8.3%, higher among females than among males (13.7% vs 3.1%). There was also a higher incidence of depressive symptoms among baseline current smokers compared to non-current smokers (17.6% vs 8.2%). However, this difference was confined to males (20.0% vs 2.9%). Consequently, smoking at baseline was associated with a higher risk of depressive symptoms only among males in all models (in fully adjusted model OR = 12.7, CI 95% 2.5–63.9, *p* = 0.002) with a statistically significant interaction between sex and current smoking (*p* = 0.005) (data not shown).

**Table 3:** Odds ratios and 95% CI of onset of depressive symptoms at follow-up according to tobacco use at baseline among students below the threshold for depressive symptoms at baseline, by sex and type of tobacco, the Kupol study 2013–2015

	*n*	Depressive symptoms cases	Unadjusted model		Adjusted model A		Adjusted model B	
			OR (95% CI)	*p* value	OR (95% CI)	*p* value	OR (95% CI)	*p* value
Current cigarette smoking								
Total students								
Yes	34	6	2.4 (1.0,5.9)	0.053	1.7 (0.6,4.3)	0.294	2.0 (0.7,5.8)^a^	0.189
No	2865	234						
Males								
Yes	15	3	8.3 (2.3,30.5)	0.001	7.3 (1.9,28.2)	0.004	12.7 (2.5,63.9)	0.002
No	1470	43						
Females								
Yes	19	3	1.2 (0.3,4.1)	0.792	0.8 (0.2,2.8)	0.682	1.0 (0.3,3.9)	0.987
No	1395	191						
Current snus use								
Total students								
Yes	22	1	0.5 (0.0,3.9)	0.531	0.4 (0.1,3.5)	0.432	0.8 (0.1,7.5)^a^	0.833
No	2878	239						
Males								
Yes	20	1	1.7 (0.2,16.7)	0.624	1.3 (0.2,10.4)	0.802	1.3 (0.2,11.6)	0.792
No	1466	45						
Females								
Yes	2	0	–		–		–	
No	1412	194						
Current tobacco use								
Total students								
Yes	50	6	1.5 (0.6,3.6)	0.338	1.1 (0.5,2.8)	0.798	1.5 (0.6,4.1)^a^	0.398
No	2850	234						
Males								
Yes	30	3	3.7 (1.1,12.5)	0.039	2.8 (0.8,10.0)	0.112	3.6 (0.9,14.8)	0.069
No	1456	43						
Females								
Yes	20	3	1.1 (0.3,3.8)	0.867	0.8 (0.2,2.7)	0.664	1.0 (0.3,3.8)	0.985
No	1394	191						
Self-reported tobacco dependence								
Total students								
Yes	29	7	3.6 (1.5,8.6)	0.003	2.5 (1.0,6.4)	0.055	4.8 (1.7,14.0)^a^	0.004
No	2827	228						
Males								
Yes	21	5	10.7 (3.7,30.6)	< 0.001	7.5 (2.4,23.3)	< 0.001	12.8 (3.5,46.9)	< 0.001
No	1444	41						
Females								
Yes	8	2	2.1 (0.4,10.6)	0.356	1.1 (0.2,6.0)	0.897	1.2 (0.2,6.7)	0.801
No	1383	187						

^a^Model A adjusted for CES-DC score at baseline. Model B as model A further adjusted for alcohol consumption, parental education, parental birthplace and sex

Current snus use (*n* = 22) or tobacco use (*n* = 50) at baseline were not associated with the odds of depressive symptoms at follow-up.

Self-reported tobacco dependence at baseline was associated with a higher odds of depressive symptoms at follow-up in the whole cohort and among males but not among females, both before and after adjustment. The interaction between sex and tobacco dependence was statistically significant (*p* = 0.017) in the fully adjusted regression model (not shown in the table).

### Association between onset of tobacco use and onset depressive symptoms between baseline and follow-up (Table 4) (step 3)

For this analysis the study sample was restricted to 2831 participants who at baseline scored below the depressive symptoms score threshold of 30 and reported no current use of any tobacco. At follow-up, the cumulative incidence of current cigarette smoking use, snus use, any tobacco use and perceived tobacco dependence in this sub-group was 2.9, 1.7, 3.9 and 1.7%, respectively. The use of snus was higher in males than females (2.9% vs 0.6%). The average incidence of depressive symptoms was 8.2%, higher among those who currently used cigarette (26.3%), snus (16.3%), any tobacco (21.5%) or who reported feeling dependent on tobacco (26.1%) (data not shown). The onset of current smoking, current snus use, current tobacco use and dependence on tobacco were associated with higher odds of developing depressive symptoms at follow-up in unadjusted and adjusted logistic regression models both in whole cohort and among males (Table 4). Among females, current smoking, current tobacco use and dependence on tobacco, but not current snus use, were associated with higher odds of depressive symptoms. Despite all associations were stronger among males the interactions between sex and tobacco use variables were non-significant in the fully adjusted models (data not shown).

**Table 4:** Initiation of tobacco use and onset of depressive symptoms between baseline and follow-up among students without depressive symptoms and no use of tobacco at baseline, the Kupol study 2014–2015

	*n*	Depressive symptoms cases	Unadjusted model		Adjusted model A		Adjusted model B	
			OR (95% CI)	*p* value	OR (95% CI)	*p* value	OR (95% CI)	*p* value
Current cigarette smoking								
Total students								
Yes	80	21	4.3 (2.6–7.2)	< 0.001	3.5 (2.0–6.2)	< 0.001	3.9 (2.1–7.2)^a^	< 0.001
No	2751	210						
Males								
Yes	39	6	7.1 (2.8–18.1)	< 0.001	5.7 (2.1–15.2)	0.001	6.7 (2.5–18.2	< 0.001
No	1404	35						
Females								
Yes	41	15	3.9 (2.0–7.4)	< 0.001	3.2 (1.6–6.6)	0.001	3.1 (1.5–6.4)	0.003
No	1347	175						
Current snus use								
Total students								
Yes	49	8	2.2 (1.0–4.8)	0.040	2.4 (1.0–5.5)	0.041	5.1 (2.1–12.8)^a^	< 0.001
No	2782	223						
Males								
Yes	41	6	6.7 (2.6–19.9)	< 0.001	6.5 (2.4–17.3)	< 0.001	7.5 (2.7–20.7)	< 0.001
No	1402	35						
Females								
Yes	8	2	2.1 (0.4–10.5)	0.362	1.5 (0.3–8.7)	0.654	1.9 (0.3–12.4)	0.518
No	1380	188						
Current tobacco use								
Total students								
Yes	107	23	3.3 (2.0-–5.4)	< 0.001	2.9 (1.7–4.9)	< 0.001	3.6 (2.0–6.4)^a^	< 0.001
No	2724	208						
Males								
Yes	62	8	6.0 (2.7–13.7)	< 0.001	5.4 (2.3–12.8)	< 0.001	6.3 (2.6–15.1)	< 0.001
No	1381	133						
Females								
Yes	45	15	3.3 (1.8–6.3)	< 0.001	2.7 (1.3–5.3)	0.005	2.6 (1.3–5.4)	0.009
No	1343	175						
Self-reported tobacco dependence								
Total students								
Yes	46	12	4.1 (2.1–8.1)	< 0.001	3.2 (1.5–6.8)	0.002	3.7 (1.7–8.2)^a^	0.001
No	2740	216						
Males								
Yes	20	2	3.9 (0.9–17.7)	0.071	3.2 (0.7–15.9)	0.147	4.2 (0.8–21.2)	0.079
No	1392	38						
Females								
Yes	26	10	4.1 (1.8–9.2)	0.001	3.2 (1.3–7.9)	0.009	3.8 (1.5–9.3)	0.004
No	1348	178						

^a^Model A adjusted for CES-DC score at baseline. Model B as model A further adjusted for alcohol consumption, parental education, parental birthplace and sex

### Association of current tobacco use at baseline and of current tobacco use at follow-up with the incidence of internalizing problems at follow-up (Supplementary Tables 1, 2, 3, 4) (step 4)

In this analysis we included 2807 students without high internalizing problems at baseline according to the self-reported SDQ subscales (cut-off score = 9). The incidence of internalizing problems at 1-year follow-up was 9.8% (4.4% and 15.2% in males and females, respectively). Current cigarette smoking and dependence on tobacco were associated with higher odds of developing internalizing symptoms at follow-up with a greater effect in females than males (Supplementary Table 1). Also considering parent-reported SDQ we found similar results (Supplementary Table 2). The study sample was then restricted to 2728 participants who not only scored below the threshold of 9 for internalizing symptoms in the SDQ, but also reported no current use of any tobacco at baseline. Reporting current tobacco at follow-up was associated with higher risk of onset of internalizing symptoms only in males. Dependence on tobacco was associated with the onset of symptoms, significantly so in the whole cohort and among girls (Supplementary Table 3). However, there were no differences in ORs magnitude between females and males and the interaction with sex was not significant. Regarding parent-reported SDQ, very few students had high internalizing problems and we could not observe any association between tobacco use and the risk of developing internalizing symptoms (Supplementary Table 4).

## Discussion

In our study we found a positive association between tobacco use at baseline and increased depressive symptoms score or onset of depressive symptoms after 1 year even after controlling for potential confounders. We also detected an association between concurrent initiation of tobacco use and onset of depressive symptoms at follow-up.

Taken together these findings strengthen the hypothesis of a causal role of tobacco in determining the onset of depressive symptoms. The effect of tobacco of cigarette and snus on depressive symptoms is probably mediated by nicotine, its primary additive constituent [18]. Nicotine may lead to an alteration of hypothalamic–pituitary–adrenal axis through an activation of central nicotinic receptors with a subsequent increase of the cortisol response [7] and an inhibition effect on the monoamine oxidase enzyme system [19]. This hypersecretion of cortisol and alteration of the neurotransmitters pathway may result in a dysregulation of neurobiological system with the subsequent onset of depressive moods. However, the findings in this study do not help ruling out the role of shared causes between progression in tobacco use/dependence and depressive symptoms. In fact, the associations of concurrent change in smoking status and depressive symptoms during the follow-up is compatible with a causal role of tobacco in determining depressive symptoms or shared risk factors between the two conditions or an inverse relationship, i.e., depressed mood causing the initiation to tobacco use.

The above mentioned review by Fluharty et al. [4] showed inconsistent results among studies evaluating the longitudinal association between tobacco use and the subsequent onset of depressive symptoms in adolescents. However, these studies were heterogeneous regarding follow-up length and instruments used for measuring depressive symptoms. Among the nine studies included in this review where the CES-DC scale was used for the assessment of depressive symptoms, seven [5, 13, 14, 20–23] reported results similar to ours, with a positive association between tobacco smoking and the subsequent onset of depressive symptoms. The two studies [15, 24] that found no association were re-analyses of the National Longitudinal Study of Adolescent Health (Add Health), and described no association using the full models adjusted for “unobservable factors” [24] and for important potential confounders [15] such as ethnicity, parental smoking, peer smoking, alcohol consumption, delinquency score and baseline depressive score. However, three studies based on the same data found a positive association even after multiple adjustments [5, 20, 21].

We primarily used the CES-DC scale as the instrument for measuring depressive symptoms as it was done in cited previous studies [5, 13–15]. At odds with other studies, we analyzed CES-DC scores as both dichotomous and continuous variables, which yielded similar associations. This was reassuring, because we believe that the use of scales’ conventional thresholds alone, however established may limit the detection of important associations at pre-clinical stages of mental ill health, while these would be better revealed by studying the full score distribution. Our results also provided support for similar associations for the use of the Swedish smokeless tobacco “snus”. However, this similarity was limited to the strong association between concurrent onset of snus use and of depressive symptoms during the year following inception. This pattern can be interpreted in different ways. On the one side, it would be difficult to establish the direction and temporality of the association, making it impossible to exclude that the onset of depressive symptoms leads to tobacco use as a self-medication strategy. On the other side, it could also indicate very short latency between exposure to tobacco or escalation of its use and depressive symptoms. This latter explanation is plausible because snus users are exposed to high and sustained levels of rapidly absorbed nicotine. The Swedish-type moist snuff is added with chemical buffering agents (sodium carbonate) [18] in order to increase the pH to around 8, thus facilitating the uptake of uncharged nicotine in the mouth [25]. At pH > 8.0, indeed, the majority of nicotine is in its free uncharged form, which would more easily pass through the lung membrane, with higher blood and brain nicotine levels than for cigarette smoking [25]. Thus, the concurrent association between onset of snus use and of depressive symptoms could indicate a causal effects of high and sustained exposure to nicotine.

We also found a consistent association between perceived tobacco dependence and the incidence of depressive symptoms at 1-year follow-up, with a stronger effect among males. Studies in the past two decades strongly suggest that tobacco dependence in adolescence may appear even with occasional use [26]. Nicotine-dependent youths might experience abstinence symptoms and frequent negative moods when craving for tobacco. It is therefore possible that at least part of the higher incidence of depressive mood among young tobacco users compared to non-users is due to withdrawal from nicotine rather than depressive disorders.

The role of sex in the association between smoking and depressive symptoms is a matter of speculations. We found somewhat unexpected consistently stronger associations among males, while prior analyses suggested a stronger relationship among girls [21, 24], as we also hypothesized base on available studies on both animals and humans [9, 27]. In fact, human and rat females are more vulnerable to neurologic and psychological consequences of cigarette smoking than males [8, 28, 29]. They generally report higher mood modifications in connection with smoking [9] and were found to be more sensitive than males to the rewarding effects of nicotine [29, 30]. Also, the onset of dependence among adolescent females seems to be more rapid than among males, after initial episodes of smoking [31, 32]. There are two possible explanations of this unexpected pattern. Firstly, it may reflect an age-related delay of effects among males. The strongest effect of tobacco use on the onset of depressive symptoms may be exerted during sexual maturation, in particular for females in connection with menarche; indeed in female adolescence rats, ovarian hormones modulate the sensitivity to nicotine’s reinforcing effects [32]. At the age of 13–14 (inception in the cohort) it is likely that a substantial higher proportion of females than of males in this cohort had already entered puberty, therefore having left behind the period of highest vulnerability to the purported depressive effects of tobacco. Secondly, if misclassification of depressive symptoms would be differential between sexes, with a higher proportion of false positives among females than among males the resulting association would be biased towards the null among the former rather than among the latter. This explanation is not lacking rationale: (1) 9 out of 20 of CES-DC items may coincide with premenstrual symptoms [33] and (2) CES-DC focuses on symptoms in the 7 days before the survey, a period short enough to be casually coinciding with pre-menstrual phase for many girls. To corroborate this possibility, there were no sex differences in the sensitivity analyses using SDQ internalizing score, which at odds with CES-DC assesses feelings during the previous 6 months and encompasses only 1 out of 10 items that could be related to premenstrual symptoms [33]. Therefore, we cannot exclude that the lack of evidence in females might be due to a low specificity of CES-DC towards depressive symptoms.

Last but not least it is important to consider the role of the social context on these associations. The Swedish context is different from that of the other countries where most studies on tobacco and depressive symptoms in adolescents were carried out, in that a particularly high rate of mental health problems among adolescents is paralleled by relatively low rates of smoking. When the important proportion of “social smokers” declines individuals taking up smoking may represent a minority of adolescents with high propensity to the disruptive neuro-biological effects of tobacco, including dependence and possibly depressive symptoms. Regarding depressive symptoms, there was a mean increase in CES-DC scores from 14.3 to 15.7 in 1-year follow-up. This is to be compared with the large USA Add Health sample where, this increase was lower, from 10.0 to 10.7 in 1 year [5]. Furthermore, in this study the mean CES-DC scores in current and non-current smokers, after 1 year, were 27.1 and 14.1, respectively, whereas they were lower in the Add Health sample, 13.0 and 9.6, respectively [20].

The current study used longitudinal data from the largest ad hoc cohort of adolescents in Sweden, the Kupol study. The Kupol study cohort provides a unique platform to evaluate the association between different student factors and mental health among adolescents. Moreover, this study benefits from: (1) the use of standardized instruments for the assessment of mental health problems and tobacco use; (2) the short time between the baseline and follow-up assessment; (3) the same age of students at the enrolment in the cohort. Also there was a high retention rate (97.7%) from baseline to 1-year follow-up, therefore the risk of attrition bias was low.

The main limitation of this study is the initial low participation rate among invited students, which may have contributed to a greater than intended inclusion of high socio-economic status participants. This selection imposes caution in generalizing the results of this study to the source adolescent population.

## Conclusion

In conclusion, we found a longitudinal association between tobacco use at baseline and short-term onset of depressive symptoms in adolescents with gender differences. The psychological and/or biological mechanisms underlying this association should be the focus of additional research.

## Electronic supplementary material

Below is the link to the electronic supplementary material.

Supplementary material 1 (DOCX 38 kb)
