# Supplementary material for: Longitudinal association between tobacco use and the onset of depressive symptoms among Swedish adolescents: the Kupol cohort study
Source: Eur Child Adolesc Psychiatry. 2018 Oct 12;28(5):695–704. doi: 10.1007/s00787-018-1237-6 (PMC6514114; doi:10.1007/s00787-018-1237-6)
Supplement: Supplementary file 1 — Supplementary material 1 (DOCX 38 kb) [file 787_2018_1237_MOESM1_ESM.docx]

**Supplementary table 1. Odds ratios and 95% CI of incidence of SDQ-assessed internalizing symptoms at follow-up according to tobacco use at baseline among students scoring below the threshold for high internalizing symptoms at baseline the Kupol study 2013-2015.**

|  |  |  |  |  | **Unadjusted model** | |  | **Adjusted model A** | |  | **Adjusted model B** | |
| --- | --- | --- | --- | --- | --- | --- | --- | --- | --- | --- | --- | --- |
|  |  | **n** | **cases** |  | **OR (95% CI)** | **p-value** |  | **OR (95% CI)** | **p-value** |  | **OR (95% CI)** | **p-value** |
| **CURRENT**  **CIGARETTE SMOKING** |  |  |  |  |  |  |  |  |  |  |  |  |
| **Total students** | Yes | 42 | 10 |  | 3.0(1.4-6.1) | 0.003 |  | 2.3(1.1-5.0) | 0.033 |  | 1.9(0.8-4.6)^a^ | 0.174 |
|  | No | 2755 | 263 |  |  |  |  |  |  |  |  |  |
|  |  |  |  |  |  |  |  |  |  |  |  |  |
| **Males** | Yes | 13 | 1 |  | 1.8(0.2-14.3) | 0.565 |  | 1.5(0.2-12.4) | 0.707 |  | 1.7(0.2-17.1) | 0.673 |
|  | No | 1399 | 61 |  |  |  |  |  |  |  |  |  |
|  |  |  |  |  |  |  |  |  |  |  |  |  |
| **Females** | Yes | 29 | 9 |  | 2.6(1.2-5.7) | 0.021 |  | 2.1(0.9-5.0) | 0.078 |  | 2.0(0.7-5.3) | 0.187 |
|  | No | 1356 | 202 |  |  |  |  |  |  |  |  |  |
|  |  |  |  |  |  |  |  |  |  |  |  |  |
| **CURRENT SNUS USE** |  |  |  |  |  |  |  |  |  |  |  |  |
| **Total students** | Yes | 22 | 0 |  | - |  |  | - |  |  | - |  |
|  | No | 2776 | 274 |  |  |  |  |  |  |  |  |  |
|  |  |  |  |  |  |  |  |  |  |  |  |  |
| **Males** | Yes | 19 | 0 |  | - |  |  | - |  |  | - |  |
|  | No | 1394 | 63 |  |  |  |  |  |  |  |  |  |
|  |  |  |  |  |  |  |  |  |  |  |  |  |
| **Females** | Yes | 3 | 0 |  | - |  |  | - |  |  | - |  |
|  | No | 1382 | 211 |  |  |  |  |  |  |  |  |  |
|  |  |  |  |  |  |  |  |  |  |  |  |  |
| **CURRENT TOBACCO USE** |  |  |  |  |  |  |  |  |  |  |  |  |
| **Total students** | Yes | 60 | 10 |  | 1.9(0.9-3.7) | 0.075 |  | 1.5(0.7-3.1) | 0.286 |  | 1.3(0.6-3.1)^a^ | 0.498 |
|  | No | 2738 | 264 |  |  |  |  |  |  |  |  |  |
|  |  |  |  |  |  |  |  |  |  |  |  |  |
| **Males** | Yes | 29 | 1 |  | 0.8(0.1-5.7) | 0.791 |  | 0.6(0.1-4.8) | 0.656 |  | 0.6(0.1-5.3) | 0.675 |
|  | No | 1384 | 62 |  |  |  |  |  |  |  |  |  |
|  |  |  |  |  |  |  |  |  |  |  |  |  |
| **Females** | Yes | 31 | 9 |  | 2.3(1.1-5.1) | 0.036 |  | 1.9(0.8-4.3) | 0.135 |  | 1.7(0.6-4.4) | 0.309 |
|  | No | 1354 | 202 |  |  |  |  |  |  |  |  |  |
|  |  |  |  |  |  |  |  |  |  |  |  |  |
| **SELF-REPORTED TOBACCO DEPENDENCE** |  |  |  |  |  |  |  |  |  |  |  |  |
| **Total students** | Yes | 35 | 7 |  | 2.3(1.0-5.4) | 0.046 |  | 1.6(0.7-3.9) | 0.311 |  | 1.3(0.5-3.7)^a^ | 0.579 |
|  | No | 2720 | 262 |  |  |  |  |  |  |  |  |  |
|  |  |  |  |  |  |  |  |  |  |  |  |  |
| **Males** | Yes | 16 | 1 |  | 1.4(0.2-11.1) | 0.728 |  | 1.1(0.1-8.8) | 0.936 |  | 1.0(0.1-9.3) | 0.996 |
|  | No | 1376 | 61 |  |  |  |  |  |  |  |  |  |
|  |  |  |  |  |  |  |  |  |  |  |  |  |
| **Females** | Yes | 19 | 6 |  | 2.6(1.0-7.0) | 0.053 |  | 1.8(0.6-5.0) | 0.266 |  | 1.4(0.5-4.5) | 0.546 |
|  | No | 1344 | 201 |  |  |  |  |  |  |  |  |  |

**Model A** adjusted for SDQ score at baseline as dichotomous variable (7-8 “slightly raised” vs 0-6 “close to average”) . **Model B** as model A further adjusted for alcohol consumption, parental education, parental birthplace and sex (^a^)

**Supplementary table 2. Odds ratios and 95% CI of incidence of parental SDQ-assessed internalizing symptoms at follow-up according to tobacco use at baseline among students scoring below the threshold for high parental SDQ-assessed internalizing symptoms at baseline the Kupol study 2013-2015.**

|  |  |  |  |  | **Unadjusted model** | |  | **Adjusted model A** | |  | **Adjusted model B** | |
| --- | --- | --- | --- | --- | --- | --- | --- | --- | --- | --- | --- | --- |
|  |  | **n** | **cases** |  | **OR (95% CI)** | **p-value** |  | **OR (95% CI)** | **p-value** |  | **OR (95% CI)** | **p-value** |
| **CURRENT**  **CIGARETTE SMOKING** |  |  |  |  |  |  |  |  |  |  |  |  |
| **Total students** | Yes | 45 | 5 |  | 2.8(1.1-7.3) | 0.032 |  | 3.0(1.1-8.1) | 0.033 |  | 2.8(0.9-8.5)^a^ | 0.078 |
|  | No | 2474 | 105 |  |  |  |  |  |  |  |  |  |
|  |  |  |  |  |  |  |  |  |  |  |  |  |
| **Males** | Yes | 13 | 2 |  | 6.6(1.4-31.1) | 0.017 |  | 7.5(1.4-39.1) | 0.016 |  | 10.5(1.6-67.5) | 0.013 |
|  | No | 1198 | 32 |  |  |  |  |  |  |  |  |  |
|  |  |  |  |  |  |  |  |  |  |  |  |  |
| **Females** | Yes | 32 | 3 |  | 1.7(0.5-5.7) | 0.388 |  | 1.8(0.5-6.3) | 0.373 |  | 1.6(0.4-6.7) | 0.490 |
|  | No | 1276 | 73 |  |  |  |  |  |  |  |  |  |
|  |  |  |  |  |  |  |  |  |  |  |  |  |
| **CURRENT SNUS USE** |  |  |  |  |  |  |  |  |  |  |  |  |
| **Total students** | Yes | 16 | 2 |  | 3.2(0.7-14.1) | 0.130 |  | 3.0(0.6-14.6) | 0.182 |  | 3.1(0.5-17.1)^a^ | 0.205 |
|  | No | 2504 | 108 |  |  |  |  |  |  |  |  |  |
|  |  |  |  |  |  |  |  |  |  |  |  |  |
| **Males** | Yes | 13 | 1 |  | 2.9(0.4-23.3) | 0.306 |  | 3.0(0.3-26.6) | 0.318 |  | 3.0(0.3-32.4) | 0.371 |
|  | No | 1199 | 33 |  |  |  |  |  |  |  |  |  |
|  |  |  |  |  |  |  |  |  |  |  |  |  |
| **Females** | Yes | 3 | 1 |  | 8.2(0.7-91.5) | 0.087 |  | 5.6(0.4-81.8) | 0.210 |  | 4.4(0.3-71.6) | 0.294 |
|  | No | 1305 | 75 |  |  |  |  |  |  |  |  |  |
|  |  |  |  |  |  |  |  |  |  |  |  |  |
| **CURRENT TOBACCO USE** |  |  |  |  |  |  |  |  |  |  |  |  |
| **Total students** | Yes | 56 | 6 |  | 2.7(1.1-6.5) | 0.024 |  | 3.1(1.2-7.6) | 0.016 |  | 3.0(1.1-8.2)^a^ | 0.033 |
|  | No | 2464 | 104 |  |  |  |  |  |  |  |  |  |
|  |  |  |  |  |  |  |  |  |  |  |  |  |
| **Males** | Yes | 22 | 3 |  | 5.9(1.7-21.0) | 0.006 |  | 7.6(2.0-29.0) | 0.003 |  | 9.3(2.2-39.3) | 0.002 |
|  | No | 1190 | 31 |  |  |  |  |  |  |  |  |  |
|  |  |  |  |  |  |  |  |  |  |  |  |  |
| **Females** | Yes | 34 | 3 |  | 1.6(0.5-5.3) | 0.451 |  | 1.7(0.5-5.9) | 0.416 |  | 1.5(0.4-6.2) | 0.549 |
|  | No | 1274 | 73 |  |  |  |  |  |  |  |  |  |
|  |  |  |  |  |  |  |  |  |  |  |  |  |
| **SELF-REPORTED TOBACCO DEPENDENCE** |  |  |  |  |  |  |  |  |  |  |  |  |
| **Total students** | Yes | 37 | 6 |  | 4.4(1.8-10.8) | 0.001 |  | 4.2(1.6-10.9) | 0.004 |  | 3.9(1.4-11.2)^a^ | 0.012 |
|  | No | 2450 | 103 |  |  |  |  |  |  |  |  |  |
|  |  |  |  |  |  |  |  |  |  |  |  |  |
| **Males** | Yes | 12 | 1 |  | 3.0(0.4-23.7) | 0.300 |  | 3.1(0.3-27.4) | 0.313 |  | 2.7(0.2-30.2) | 0.424 |
|  | No | 1180 | 32 |  |  |  |  |  |  |  |  |  |
|  |  |  |  |  |  |  |  |  |  |  |  |  |
| **Females** | Yes | 24 | 5 |  | 4.4(1.6-12.2) | 0.004 |  | 4.1(1.4-12.2) | 0.011 |  | 4.1(1.3-13.5) | 0.018 |
|  | No | 1270 | 71 |  |  |  |  |  |  |  |  |  |

**Model A** adjusted for parental SDQ score at baseline as dichotomous variable (6-7 “slightly raised” vs 0-5 “close to average”). **Model B** as model A further adjusted for alcohol consumption, parental education, parental birthplace and sex (^a^).

**Supplementary table 3. Initiation of tobacco use and onset of SDQ-assessed internalizing symptoms between baseline and follow-up he Kupol study 2014-2015 among students without high internalizing symptoms at baseline and no use of tobacco.**

|  |  |  |  |  | **Unadjusted model** | |  | **Adjusted model A** | |  | **Adjusted model B** | |
| --- | --- | --- | --- | --- | --- | --- | --- | --- | --- | --- | --- | --- |
|  |  | **n** | **cases** |  | **Coeff. (95% CI)** | **p-value** |  | **Coeff. (95% CI)** | **p-value** |  | **Coeff. (95% CI)** | **p-value** |
| **CURRENT**  **CIGARETTE SMOKING** |  |  |  |  |  |  |  |  |  |  |  |  |
| **Total students** | Yes | 79 | 10 |  | 1.4(0.7-2.7) | 0.357 |  | 1.4(0.7-2.7) | 0.390 |  | 1.2(0.6-2.6)^a^ | 0.578 |
|  | No | 2641 | 252 |  |  |  |  |  |  |  |  |  |
|  |  |  |  |  |  |  |  |  |  |  |  |  |
| **Males** | Yes | 38 | 4 |  | 2.6(0.9-7.5) | 0.081 |  | 3.0(1.0-8.9) | 0.046 |  | 3.2(1.1-9.5) | 0.038 |
|  | No | 1336 | 58 |  |  |  |  |  |  |  |  |  |
|  |  |  |  |  |  |  |  |  |  |  |  |  |
| **Females** | Yes | 41 | 6 |  | 1.0(0.4-2.4) | 0.967 |  | 0.9(0.4-2.2) | 0.780 |  | 0.8(0.3-2.1) | 0.621 |
|  | No | 1305 | 194 |  |  |  |  |  |  |  |  |  |
|  |  |  |  |  |  |  |  |  |  |  |  |  |
| **CURRENT SNUS USE** |  |  |  |  |  |  |  |  |  |  |  |  |
| **Total students** | Yes | 48 | 6 |  | 1.3(0.6-3.2) | 0.498 |  | 1.6(0.7-3.9) | 0.282 |  | 2.7(1.1-6.8)^a^ | 0.035 |
|  | No | 2672 | 256 |  |  |  |  |  |  |  |  |  |
|  |  |  |  |  |  |  |  |  |  |  |  |  |
| **Males** | Yes | 39 | 4 |  | 2.5(0.9-7.3) | 0.090 |  | 2.9(1.0-8.7) | 0.051 |  | 3.0(1.0-8.9) | 0.052 |
|  | No | 1335 | 58 |  |  |  |  |  |  |  |  |  |
|  |  |  |  |  |  |  |  |  |  |  |  |  |
| **Females** | Yes | 9 | 2 |  | 1.6(0.3-8.0) | 0.537 |  | 1.6(0.3-8.2) | 0.579 |  | 2.2(0.4-12.3) | 0.383 |
|  | No | 1337 | 198 |  |  |  |  |  |  |  |  |  |
|  |  |  |  |  |  |  |  |  |  |  |  |  |
| **CURRENT TOBACCO USE** |  |  |  |  |  |  |  |  |  |  |  |  |
| **Total students** | Yes | 106 | 14 |  | 1.5(0.8-2.6) | 0.206 |  | 1.5(0.8-2.7) | 0.208 |  | 1.6(0.8-3.0)^a^ | 0.167 |
|  | No | 2614 | 248 |  |  |  |  |  |  |  |  |  |
|  |  |  |  |  |  |  |  |  |  |  |  |  |
| **Males** | Yes | 60 | 6 |  | 2.5(1.0-6.0) | 0.043 |  | 2.9(1.2-7.0) | 0.022 |  | 3.1(1.2-7.6) | 0.015 |
|  | No | 1314 | 56 |  |  |  |  |  |  |  |  |  |
|  |  |  |  |  |  |  |  |  |  |  |  |  |
| **Females** | Yes | 46 | 8 |  | 1.2(0.6-2.6) | 0.624 |  | 1.1(0.5-2.4) | 0.868 |  | 1.0(0.4-2.4) | 0.936 |
|  | No | 1300 | 192 |  |  |  |  |  |  |  |  |  |
|  |  |  |  |  |  |  |  |  |  |  |  |  |
| **SELF-REPORTED** **TOBACCO DEPENDENCE** |  |  |  |  |  |  |  |  |  |  |  |  |
| **Total students** | Yes | 46 | 10 |  | 2.7(1.3-5.4) | 0.007 |  | 3.0(1.4-6.2) | 0.004 |  | 3.0(1.3-6.5)^a^ | 0.007 |
|  | No | 2628 | 249 |  |  |  |  |  |  |  |  |  |
|  |  |  |  |  |  |  |  |  |  |  |  |  |
| **Males** | Yes | 19 | 2 |  | 2.5(0.6-11.2) | 0.223 |  | 3.2(0.7-14.1) | 0.131 |  | 3.1(0.7-14.3) | 0.144 |
|  | No | 1323 | 59 |  |  |  |  |  |  |  |  |  |
|  |  |  |  |  |  |  |  |  |  |  |  |  |
| **Females** | Yes | 27 | 8 |  | 2.5(1.1-5.7) | 0.035 |  | 2.6(1.1-6.3) | 0.030 |  | 2.8(1.1-7.0) | 0.028 |
|  | No | 1305 | 190 |  |  |  |  |  |  |  |  |  |

**Model A** adjusted for SDQ score at baseline as dichotomous variable (7-8 “slightly raised” vs 0-6 “close to average”). **Model B** as model A further adjusted for alcohol consumption, parental education, and parental birthplace and sex (^a^).

**Supplementary table 4. Initiation of tobacco use and onset of parental SDQ-assessed internalizing symptoms between baseline and follow-up he Kupol study 2014-2015 among students without high parental SDQ-assessed internalizing symptoms at baseline and no use of tobacco.**

|  |  |  |  |  | **Unadjusted model** | |  | **Adjusted model A** | |  | **Adjusted model B** | |
| --- | --- | --- | --- | --- | --- | --- | --- | --- | --- | --- | --- | --- |
|  |  | **n** | **cases** |  | **Coeff. (95% CI)** | **p-value** |  | **Coeff. (95% CI)** | **p-value** |  | **Coeff. (95% CI)** | **p-value** |
| **CURRENT**  **CIGARETTE SMOKING** |  |  |  |  |  |  |  |  |  |  |  |  |
| **Total students** | Yes | 81 | 2 |  | 0.6(0.1-2.4) | 0.451 |  | 0.5(0.1-2.3) | 0.417 |  | 0.5(0.1-2.2)^a^ | 0.359 |
|  | No | 2365 | 99 |  |  |  |  |  |  |  |  |  |
|  |  |  |  |  |  |  |  |  |  |  |  |  |
| **Males** | Yes | 35 | 1 |  | 1.1(0.1-8.3) | 0.930 |  | 1.0(0.1-7.9) | 0.991 |  | 1.3(0.2-10.7) | 0.792 |
|  | No | 1147 | 31 |  |  |  |  |  |  |  |  |  |
|  |  |  |  |  |  |  |  |  |  |  |  |  |
| **Females** | Yes | 46 | 1 |  | 0.4(0.1-2.7) | 0.329 |  | 0.4(0.0-2.8) | 0.328 |  | 0.3(0.0-2.6) | 0.287 |
|  | No | 1218 | 69 |  |  |  |  |  |  |  |  |  |
|  |  |  |  |  |  |  |  |  |  |  |  |  |
| **CURRENT SNUS USE** |  |  |  |  |  |  |  |  |  |  |  |  |
| **Total students** | Yes | 43 | 1 |  | 0.5(0.1-4.0) | 0.555 |  | 0.7(0.1-5.6) | 0.769 |  | 1.0(0.1-7.4)^a^ | 0.981 |
|  | No | 2403 | 100 |  |  |  |  |  |  |  |  |  |
|  |  |  |  |  |  |  |  |  |  |  |  |  |
| **Males** | Yes | 31 | 1 |  | 1.2(0.2-9.4) | 0.832 |  | 1.6(0.2-12.8) | 0.657 |  | 1.8(0.2-14.9) | 0.581 |
|  | No | 1151 | 30 |  |  |  |  |  |  |  |  |  |
|  |  |  |  |  |  |  |  |  |  |  |  |  |
| **Females** | Yes | 12 | 0 |  | - |  |  | - |  |  | - |  |
|  | No | 1252 | 70 |  |  |  |  |  |  |  |  |  |
|  |  |  |  |  |  |  |  |  |  |  |  |  |
| **CURRENT TOBACCO USE** |  |  |  |  |  |  |  |  |  |  |  |  |
| **Total students** | Yes | 102 | 2 |  | 0.5(0.1-1.9) | 0.273 |  | 0.5(0.1-2.0) | 0.299 |  | 0.5(0.1-2.0)^a^ | 0.293 |
|  | No | 2344 | 99 |  |  |  |  |  |  |  |  |  |
|  |  |  |  |  |  |  |  |  |  |  |  |  |
| **Males** | Yes | 49 | 1 |  | 0.7(0.1-5.6) | 0.779 |  | 0.8(0.1-6.1) | 0.810 |  | 1.1(0.1-8.3) | 0.957 |
|  | No | 1132 | 30 |  |  |  |  |  |  |  |  |  |
|  |  |  |  |  |  |  |  |  |  |  |  |  |
| **Females** | Yes | 52 | 1 |  | 0.3(0.0-2.4) | 0.269 |  | 0.3(0.0-2.5) | 0.286 |  | 0.3(0.0-2.4) | 0.266 |
|  | No | 1212 | 69 |  |  |  |  |  |  |  |  |  |
|  |  |  |  |  |  |  |  |  |  |  |  |  |
| **SELF-REPORTED** **TOBACCO DEPENDENCE** |  |  |  |  |  |  |  |  |  |  |  |  |
| **Total students** | Yes | 45 | 0 |  | - |  |  | - |  |  | - |  |
|  | No | 2357 | 100 |  |  |  |  |  |  |  |  |  |
|  |  |  |  |  |  |  |  |  |  |  |  |  |
| **Males** | Yes | 17 | 0 |  | - |  |  | - |  |  | - |  |
|  | No | 1136 | 31 |  |  |  |  |  |  |  |  |  |
|  |  |  |  |  |  |  |  |  |  |  |  |  |
| **Females** | Yes | 28 | 0 |  | - |  |  | - |  |  | - |  |
|  | No | 1221 | 69 |  |  |  |  |  |  |  |  |  |

**Model A** adjusted for parental SDQ score at baseline as dichotomous variable (6-7 “slightly raised” vs 0-5 “close to average”). **Model B** as model A further adjusted for alcohol consumption, parental education, and parental birthplace and sex (^a^).
